# Exploratory Immunohistochemical Profiling of FOXP3, PD-1 and CD32B in Resectable Lung Adenocarcinoma

**DOI:** 10.3390/cancers17233886

**Published:** 2025-12-04

**Authors:** Long-Wei Lin, Hong-Jing Chuang, Kuan-Hsun Lian, Yu-Ting Tseng, Chung-Yu Chen

**Affiliations:** 1 Department of Pathology, National Taiwan University Hospital Yunlin Branch, Douliu 640203, Taiwan; 2 Department of Surgery, National Taiwan University Hospital Yunlin Branch, Douliu 640203, Taiwan; 3 Department of Internal Medicine, National Taiwan University Hospital Yunlin Branch, Douliu 640203, Taiwan; 4 College of Medicine, National Taiwan University, Taipei 100233, Taiwan

**Keywords:** lung cancer, tumor microenvironment, FOXP3, PD-1, CD32B

## Abstract

**Background:** Regulatory T cells (FOXP3^+^), checkpoint signaling (PD-1), and inhibitory B-cell signaling (CD32B/FCGR2B) may shape recurrence risk after resection of lung adenocarcinoma, but small, stage-heterogeneous cohorts complicate inference. **Methods:** We profiled 21 resected lung adenocarcinomas by immunohistochemistry (IHC) for CD3, CD8, FOXP3, PD-1, CD19, and CD32B. Five systematically sampled 200× fields per stain were quantified in ImageJ to derive continuous percentages and prespecified ratios: FOXP3/CD8 and CD32B/CD19 (primary), and PD-1/CD8 (exploratory). Analyses emphasized effect sizes with exact non-parametric tests for clinicopathologic associations and Cox time-to-event models for disease-free survival (DFS). Kaplan–Meier plots used median splits for visualization only. **Results:** Higher immunosuppressive balance associated with adverse features and shorter DFS. Patients with higher FOXP3/CD8 and CD32B/CD19 had markedly shorter DFS on K-M displays (FOXP3/CD8: 18.9 vs. 45.6 months; CD32B/CD19: 25.0 vs. 72.8 months). In Cox models, each ratio was associated with increased hazard of recurrence (FOXP3+PD-1/CD8, HR 2.03, 95% CI 1.26–3.29; CD32B/CD19, HR 1.98, 95% CI 1.16–3.37). **Conclusions:** In this hypothesis-generating pilot, an immunosuppressive tumor microenvironment, indexed by higher FOXP3 (relative to CD8) and higher CD32B (relative to CD19), portends earlier recurrence after surgery. These results support external validation in larger, stage-balanced cohorts and motivate incorporation of quantitative IHC ratios into postoperative risk stratification.

## Simple Summary

This pilot study explored whether immune cells surrounding lung adenocarcinoma can help predict which patients are more likely to have their cancer return after surgery. We used immunohistochemistry to measure regulatory T-cell marker FOXP3, checkpoint protein PD-1, and inhibitory B-cell receptor CD32B in 21 resected tumors and ex-pressed them as ratios to cytotoxic T cells (CD8) or B cells (CD19). Higher FOXP3/CD8, FOXP3+PD-1/CD8, and CD32B/CD19 ratios were linked to more advanced disease features and shorter disease-free survival, suggesting a more immunosuppressive tumor microenvironment. Although our sample is small, these quantitative immune markers may complement traditional staging and deserve validation in larger studies.

## 1. Introduction

Immunotherapy represents a significant area of focus within the broader field of cancer treatment [1,2]. The immune system is constituted of two distinct branches: innate immunity and adaptive immunity. Adaptive immunity is distinguished by the activation of naïve T and B lymphocytes, which subsequently differentiate into effector T (cytotoxic T lymphocyte [CTL] and T helper [Th] cells) and B (plasma) cells, respectively. This process confers both specific and memory effects. T cells express a variety of functional proteins that are integral to immune defense and regulatory processes [3,4]. Furthermore, T cells express cytotoxic T lymphocyte-associated protein 4 (CTLA-4) and programmed cell death protein 1 (PD-1), which provide inhibitory signaling to prevent T cell activation [5,6,7,8]. In view of the foregoing, novel antibody-based therapeutics targeting CTLA-4 and PD-1 have been developed for the treatment of select cancers, including melanoma, lung, and renal cancers. Monoclonal antibodies, such as ipilimumab (Bristol Myers Squibb) and pembrolizumab (Merck) or nivolumab (Bristol Myers Squibb), are administered to cancer patients with the objective of reactivating T-cell-mediated immunity [9]. This enhances the ability of CTL and Th cells to recognize tumor antigens and eliminate tumors.

Forkhead Box P3 (FOXP3) is also a member of the forkhead/winged-helix family and plays a pivotal role in the differentiation and maturation of regulatory T cells (Treg cells), which are essential for maintaining immune self-tolerance and stability. FOXP3 serves as a negative regulator, impeding IL-2 production and, in consequence, curbing T-cell activation. Moreover, it produces immunosuppressive effects through the secretion of immunosuppressive cytokines, such as IL-10 and TGF-β, which ultimately result in immunosuppression [10,11,12,13,14,15,16,17]. Similarly, analogous molecules are expressed on the surface of B cells, providing inhibitory signaling that blocks B cell activation. Beyond T-cell checkpoints, B cells also influence anti-tumor immunity [3]. Intratumoral B cells often form tertiary lymphoid structures (TLSs), enabling antigen presentation and the generation of high-affinity tumor-specific antibodies. Well-organized TLSs in several solid tumors correlate with better outcomes and stronger immunotherapy responses [18]. However, B-cell activity is constrained by inhibitory receptors that limit excessive or autoreactive activation [19].

To illustrate, CD32B (FCGR2B) is a receptor on the B cell membrane that induces an inhibitory signal to maintain immune system homeostasis and prevent B cell activation and subsequent attack of self-antigens [20,21,22,23,24]. Consequently, the activation of CD32B (FCGR2B) represents a novel therapeutic approach for the treatment of autoimmune and inflammatory disorders. The extant research corpus lends support to the assertion that immune modulatory antibodies can interact with disparate types of FCR receptors, thereby activating and exerting their beneficial effects. For the TNFR superfamily, the interaction with multiple antibodies is necessary to activate the downstream signaling pathways [25]. In murine experiments, the inhibitory CD32B (FCGR2B) receptor has been demonstrated to be associated with the activation of numerous TNFR targets, including CD40, death receptor 5 (DR5), and CD95 [26,27,28,29]. The interaction between IgG Fc and CD32B (FCGR2B) receptors can result in the clustering and subsequent activation of TNFRs, which in turn initiates downstream signaling pathways. Conversely, the efficacy of antibodies themselves, such as antibody-dependent cellular cytotoxicity (ADCC) and antibody-dependent cellular phagocytosis (ADCP), is contingent upon the activation of various FCRs. Experiments conducted on mice have demonstrated that the activation of FCR receptors facilitates the anti-tumor effects of immune modulatory anti-OX40 and anti-GITR antibodies, enabling the selective elimination of regulatory T cells within tumors [30,31].

Recent advances in immunotherapy have highlighted the importance of immune evasion mechanisms in lung cancer. Regulatory T cells (Tregs), characterized by FOXP3 expression, and immune checkpoint proteins such as PD-1 are known to suppress anti-tumor immune responses, allowing tumor progression. Additionally, CD32B (FCGR2B), an inhibitory receptor expressed on B cells, contributes to immune suppression by downregulating B cell activation. While prior studies have investigated FOXP3 and PD-1 in the context of lung cancer, the role of CD32B remains underexplored. This study aims to elucidate the prognostic significance of these markers in resectable lung adenocarcinoma, differentiating itself from prior research by focusing on their combined impact on patient outcomes. This study is designed as a pilot to generate effect-size estimates and feasibility metrics for a larger, adequately powered validation.

## 2. Material and Methods

### 2.1. Cases and Design

We retrospectively identified 21 patients with resectable lung adenocarcinoma and sufficient archival tissue; no patient received neoadjuvant therapy. Clinical–pathologic variables were abstracted according to AJCC 8th edition, including tumor size, nodal status, pathologic stage, grade, visceral pleural invasion (PL), and spread through air spaces (STAS). The study was approved by the institutional review board (202207135RIN). The primary endpoint was disease-free survival (DFS); overall survival (OS) was secondary.

### 2.2. Immunohistochemistry and Image Acquisition

Formalin-fixed, paraffin-embedded sections (4 μm) were stained on a BOND-MAX automated immunostainer (Leica Biosystems, Nussloch, Germany) using the following primary antibodies: CD3 (clone LN10, Leica Biosystem, Nussloch, Germany, cat. no.NCL-L-CD3-565, 1:200), CD8 (clone 4B11, Leica Biosystem, Nussloch, Germany, cat. no.NCL-L-CD8-4B11, 1:100), FOXP3 (clone 259D, BioLegend, San Diego, CA, USA, cat. no. AB_430885(No.320202), 1:50), PD-1 (clone NAT105, GeneTex, Irvine, CA, USA, cat. no. GTX20256, 1:50), CD19 (clone BT51E, Leica Biosystem, Nussloch, Germany, cat. no.NCL-L-CD19-163,1:100), and CD32B (clone EP888Y, Abcam, Waltham, MA, USA, 1:2000).

Antigen retrieval was performed on a Leica BOND-MAX automated staining system using Bond Epitope Retrieval Solution 1 (ER1, pH 5.9–6.1) or Solution 2 (ER2, pH 8.9–9.1), according to the manufacturer’s instructions for each antibody. Heat-induced epitope retrieval in ER2 was carried out for 30 minutes for CD3 and PD-1, and for 20 minutes for CD8, FOXP3, and CD19, while no retrieval was applied for CD32b. All subsequent steps, including blocking, the detection system, and counterstaining, were performed in accordance with the manufacturer’s standard protocols.

Each IHC run included external positive control tissue (reactive tonsil or lymph node for T-cell and B-cell markers) processed in parallel, as well as internal positive controls within the tumor sections (tumor-infiltrating lymphocytes and stromal lymphocytes with expected staining patterns). Negative controls were performed by replacing the primary antibody with an isotype-matched control or buffer, and by confirming absence of nonspecific chromogen deposition in non-lymphoid elements (tumor epithelium, stroma). Stains were accepted only if control tissues showed the expected reactivity and negative controls were clean.

For each stain, five tumor regions were selected at 200× magnification by systematic sampling while excluding artifacts (folds, necrosis, cautery). Whole-field images were analyzed in ImageJ Version 1.54p, using the IHC Profiler plugin with fixed, a priori color-deconvolution thresholds that were applied identically across all cases and runs. For each field and marker, we obtained the percentage of DAB-positive area relative to total tissue area.

For patient-level analyses, we used the mean percentage-positive area across the five fields for each marker. From these continuous values we derived the prespecified ratios FOXP3/CD8 and CD32B/CD19 (primary) and PD-1/CD8 (exploratory). No arbitrary cut-offs were used for inferential statistics. All time-to-event models and correlation analyses were performed on continuous marker ratios, reporting hazard ratios per interquartile-range increase and exact non-parametric statistics as detailed below. Median splits were used only for Kaplan–Meier visualization. Representative control images are provided in Figure S1.

### 2.3. Image Data and Statistical Analysis

Images at 200× magnification were acquired from five systematically sampled tumor fields per stain and quantified in ImageJ Version 1.54p with fixed, a priori color-deconvolution thresholds to yield continuous percentage-positive areas; from these we derived the prespecified primary IHC ratios FOXP3/CD8 and CD32B/CD19 and the exploratory ratio PD-1/CD8. Region-of-interest (ROI) selection was performed by a thoracic pathologist, after which all image analysis steps (color deconvolution, thresholding, and percentage-area calculations) were automated using fixed macros in ImageJ/IHC Profiler Version 1.54p. The same thresholds and workflow were applied to all cases and stains. At the time of ROI selection and digital quantification, the pathologist and image analyst were blinded to clinical outcomes (recurrence status and survival). Clinical–pathologic data and outcomes were merged with the IHC dataset only after completion of the image analysis.

Given the small, stage-heterogeneous cohort (*n* = 21) and limited events, all analyses emphasized effect sizes over dichotomized tests: associations with clinicopathologic factors used exact Wilcoxon/Mann–Whitney or Kruskal–Wallis tests (with Dunn’s post-hoc where applicable) and Spearman correlations, with Benjamini–Hochberg FDR control for multiple hypotheses. The primary endpoint was DFS; OS was secondary. Time-to-event associations were modeled with bias-reduced Cox regression using continuous marker ratios and a parsimonious, prespecified adjustment set (pathologic stage [I–II vs. III] and histologic grade [G1 vs. G2–3]) to respect low events-per-variable. We reported hazard ratios per interquartile-range (IQR) increase with 95% CIs, assessed proportional hazards via Schoenfeld residuals, and explored nonlinearity with restricted cubic splines. To quantify performance and guard against overfit, we computed Harrell’s C-index and calibration slope with bootstrap optimism correction (1000 resamples); because medians can be unstable in small samples, we complemented log-rank displays with restricted mean survival time (RMST) differences at 36 months (bootstrap 95% CIs). Kaplan–Meier curves (when shown) used median splits for visualization only and were not used for inference. Two-sided α = 0.05. Scripts/macros are provided in the Supplement for reproducibility.

## 3. Results

### 3.1. Clinical and Pathological Findings of Lung Cancer Patients

The median age of the patients was 66 years, ranging from 41 to 86 years. The cohort included 12 females (57.1%) and 9 males (42.9%). Ten patients (47.6%) were current or former smokers. A majority of 14 patients (66.7%) had a tumor size greater than 3 cm. Regarding pathological stage, 7 patients (33.3%) were at Stage I-II, 8 patients (38.1%) at Stage IIIA, and 6 patients (28.5%) at Stage IIIB. The Acinar subtype was predominant, observed in 16 patients (76.1%). Pleural invasion was absent in 13 patients (61.9%). Histologically, 14 patients (66.7%) had Grade 2 (G2) tumors, while 6 patients (28.5%) had Grade 3 (G3) tumors. STAS was present in 12 patients (57.2%). Eleven (52.4%) carried an EGFR mutation, and six (28.6%) had positive PD-L1 expression (≥1%). Seventeen patients received adjuvant chemotherapy, including 11 who underwent platinum-based regimens. Disease progression occurred in 10 patients (47.6%), with 6 patients (28.5%) succumbing during the study period. The median Disease-Free Survival (DFS) was 18.97 months (range: 5.77–73.90 months), and the median Overall Survival (OS) was also 25.20 months (range: 7.03–106.63 months) (Table 1).

### 3.2. Clinicopathologic Associations of T- and B-Cell Immunomarker Expression in the Tumor Microenvironment

Table 2 presents data on the evaluation of the tumor microenvironment through immunohistochemical staining for several immune markers as T cells (CD3, CD8, FOXP3, PD1), and B cells (CD19 and CD32B). The results are stratified by tumor size (T stage), nodal involvement (*N* stage), pathological stage, histological grade, presence of spread through air spaces (STAS), and visceral pleural invasion (PL). FOXP3 expression was significantly elevated in advanced disease features, including N2 lymph node metastasis (14.77 ± 9.42, *p* < 0.05), histological grade G3 (11.69 ± 6.73, *p* < 0.05), and stage IIIB (19.72 ± 4.15, *p* < 0.05). Similarly, PD1 expression increased with tumor progression, peaking in T3 + T4 tumors (27.49 ± 8.74, *p* < 0.05) and grade G3 (26.19 ± 11.68, *p* < 0.05). CD32B was significantly higher in tumors with STAS (32.85 ± 11.34, *p* < 0.05) and visceral pleural invasion PL2 (32.64 ± 9.26, *p* < 0.05), reflecting its role in immune evasion. Notably, CD19 expression was markedly lower in poorly differentiated tumors (G3: 2.46 ± 1.03, *p* < 0.05).

### 3.3. Correlation Analysis Between the T Cells and B Cells Immunomarkers Expression

**Figure 1:**
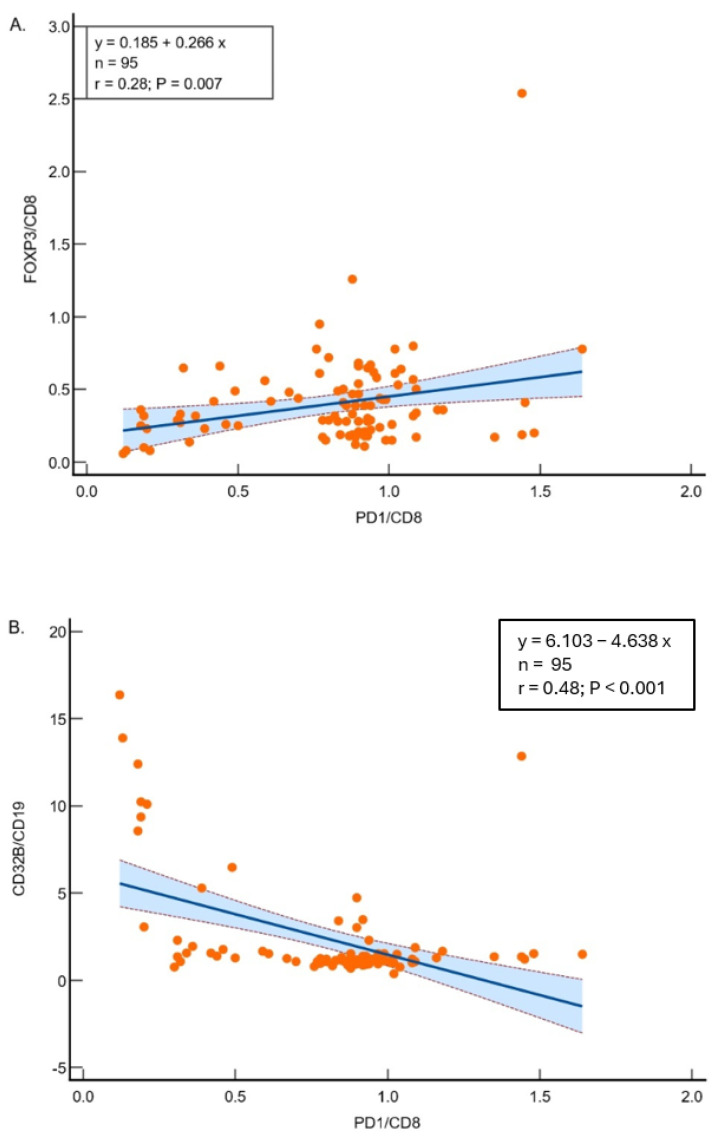
(**A**) Regression analysis between FOXP3/CD8 and PD1/CD8 ratios in 95 samples. The model demonstrates significant relationships (R^2^ = 0.07621; *p* = 0.0068), suggesting other factors influence the variability. (**B**) Regression analysis between CD32B/CD19 and PD1/CD8 ratios. The significant inverse relationship (R^2^ = 0.2313; *p* < 0.0001) highlights the interplay between these markers in lung adenocarcinoma. Scatter plots showing the correlations between FOXP3/CD8 and PD1/CD8 (**A**) and CD32B/CD19 and PD1/CD8 (**B**). Each dot represents one tumor sample. The solid line indicates the fitted linear regression, and the blue shaded area with flanking dashed lines represents the 95% confidence interval of the regression line.

The linear regression analysis showed a significant positive relationship between the ratio of FOXP3 to CD8 and the ratio of PD1 to CD8, with a slope of 0.266 (R^2^ = 0.076), and the overall regression is statistically significant (F-ratio = 7.671, *p* = 0.007) (Figure 1A). The linear regression analysis revealed a significant inverse relationship between the ratio of CD32B to CD19 and the ratio of PD1 to CD8, with a slope of −4.638 (R^2^ = 0.2313), and the overall regression is statistically significant (F-ratio = 27.984, *p* < 0.001) (Figure 1B), suggesting differing mechanisms of immune suppression.

### 3.4. The Expression of T-Cell Immunomarkers and the Clinical Correlation in Lung Adenocarcinoma

**Figure 2:**
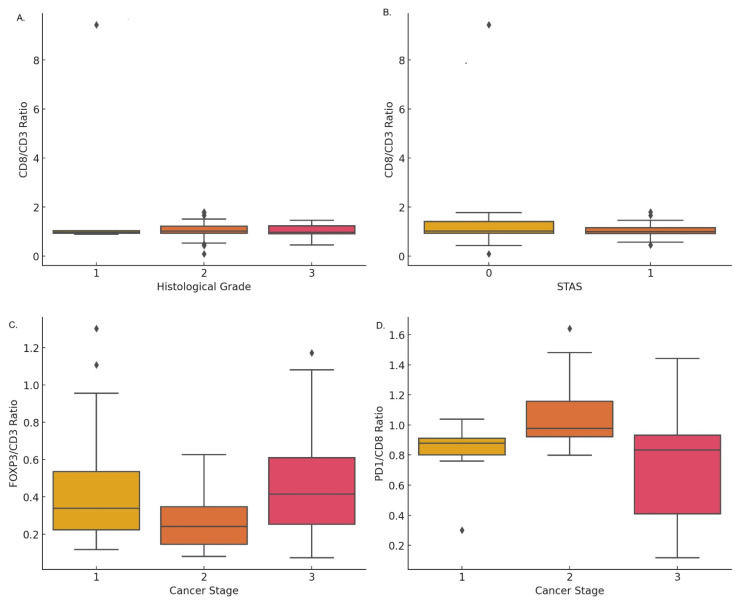
Expression of T-cell immunomarkers correlated with clinical parameters in lung cancer: (**A**) CD8/CD3 ratio across histological grades showing variability in grade 2. (**B**) CD8/CD3 ratio stratified by STAS, showing minor differences in expression levels. (**C**) FOXP3/CD3 ratio across cancer stages, with a decrease in stage 2 followed by a rise in stage 3. (**D**) PD1/CD8 ratio by cancer stage, with higher median levels in stage 2 and broader variability in stage 3.

The expression of cytotoxic T-cells, measured by the CD8/CD3 ratio, was significantly higher in lower histological grades (*p* < 0.001). The mean values were 2.65 ± 3.79 for grade 1, 1.07 ± 0.32 for grade 2, and 1.01 ± 0.28 for grade 3 (Figure 2A). Furthermore, a significant correlation was observed between the expression of CD8/CD3 and the absence of tumor spread through air spaces (STAS) (*p* = 0.001), with mean values of 1.36 ± 0.58 in cases without STAS and 3.38 ± 4.23 in cases with STAS (Figure 2B). A decreasing trend in CD8/CD3 expression was noted as cancer progressed by tumor size, lymph node involvement, visceral pleural invasion, and clinical staging, although these differences were not statistically significant.

The expression of FOXP3/CD3 increased with the progression of lung cancer pathological staging, with mean values of 0.46 ± 0.35 in stage I, 0.26 ± 0.13 in stage II, and 0.48 ± 0.26 in stage III (Figure 2C). Additionally, the expression of FOXP3/CD3 was significantly higher in cases with lymph node metastasis (*p* = 0.007), with a mean of 0.34 ± 0.26 in N0 and 0.49 ± 0.29 in N1 + 2. No significant difference was found in tumor size (T). However, FOXP3/CD3 expression was significantly higher in cases with visceral pleural invasion (*p* = 0.001), with values of 0.36 ± 0.25 in PL0 and 0.50 ± 0.31 in PL1 + 2. There was no significant correlation between FOXP3/CD3 expression and histological grade or STAS.

The FOXP3/CD8 ratio showed an increase in cytotoxic T-cell (CD8) expression with advancing cancer staging, histological grading, and the presence of lymph node or visceral pleural invasion. However, these differences were not statistically significant. Similarly, PD1 expression on T-cells, measured by the PD1/CD4 ratio, increased with cancer progression, but these differences also did not reach statistical significance.

The expression of PD1 on cytotoxic T-cells (CD8) was found to significantly increase with cancer staging (*p* < 0.001). A higher PD1/CD8 ratio was observed in stages I and II (0.83 ± 0.08 and 1.07 ± 0.24, respectively) compared to stage III (0.79 ± 0.26) (Figure 2D). Additionally, a significant correlation was observed between a higher PD1/CD8 ratio and larger tumor size (T) as well as lymph node metastasis (*N*) (*p* < 0.001). PD1/CD8 expression was also significantly higher in lower histological grades (*p* < 0.001), with mean values of 0.21 ± 0.11 for grade 1, 0.75 ± 0.27 for grade 2, and 1.33 ± 0.46 for grade 3. Lastly, PD1 expression on T-cells, measured by the PD1/CD4 ratio, was significantly higher in cases with tumor spread through air spaces (STAS) *(p* = 0.010), with values of 0.90 ± 0.17 in cases with STAS and 0.73 ± 0.41 in cases without STAS.

### 3.5. The Expression of B-Cell Immunomarkers and the Clinical Correlation in Lung Adenocarcinoma

**Figure 3:**
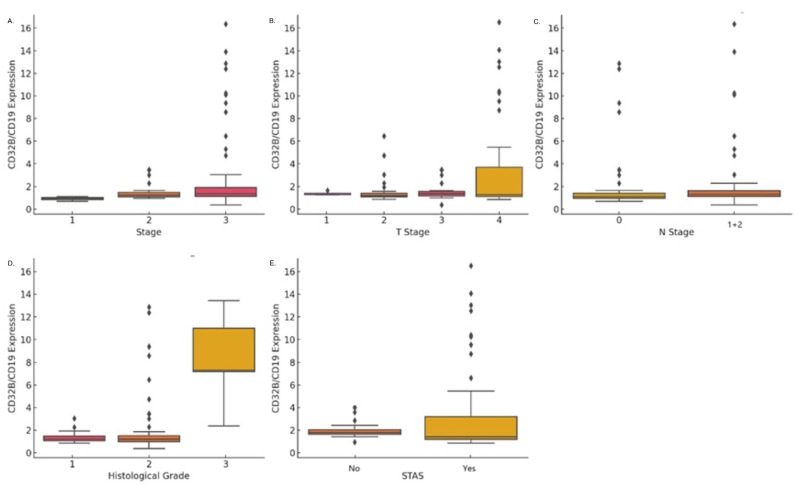
CD32B/CD19 expression across clinical and pathological factors: (**A**) Higher CD32B/CD19 expression with advancing cancer stages (*p* = 0.005). (**B**) Significantly elevated expression in T3 and T4 tumors compared to T1 and T2 (*p* = 0.006). (**C**) CD32B/CD19 expression by *N* classification (N0 vs. N1 + 2), showing no significant difference. (**D**) Higher CD32B/CD19 expression in grade 3 tumors compared to grades 1 and 2 (*p* < 0.001). (**E**) Elevated CD32B/CD19 expression in tumors with STAS compared to those without (*p* = 0.001).

The expression of CD32B has been established as a marker for B-cell (CD19) activation. The staining expression of CD32B/CD19 increased with the progression of lung cancer pathological staging. The mean expression levels in stage I and stage II were 1.27 ± 0.65, compared to 3.07 ± 3.87 in stages III (*p* = 0.005) (Figure 3A). Additionally, CD32B/CD19 expression was significantly higher in T3 and T4 tumors (*p* = 0.006), with mean values of 1.59 ± 0.73 for T1, 1.53 ± 1.11 for T2, and 3.61 ± 4.70 for T3 + T4 (Figure 3B). No significant difference was observed in the *N* classification (Figure 3C).

In terms of histological grade, CD32B/CD19 expression was significantly higher in higher grades (*p* < 0.001): grade 1 had a mean expression of 1.33 ± 0.46, compared to 2.07 ± 2.59 in grade 2, and grade 3 11.18 ± 4.21 in grade 3 (Figure 3D). CD32B/CD19 expression was also significantly higher in cases with tumor spread through air spaces (STAS) (*p* = 0.001), with mean values of 1.36 ± 0.58 in the absence of STAS, compared to 3.38 ± 4.23 when STAS was present (Figure 3E). No statistically significant correlation was found between CD32b/CD19 expression and visceral pleural invasion, or mutations in the oncoproteins EGFR and ALK.

### 3.6. Impact of Immunomarker Expression on Survival in Lung Cancer Patients

Patients exhibiting lymph node metastasis (with versus without: disease-Free Survival (DFS), median: 11.40 ± 1.56 versus 45.57 ± 9.33, *p* < 0.001), higher histologic grade (Grade 1 versus Grade 2 + 3: DFS, median, 25.3 ± 4.20 versus 11.20 ± 2.47, *p* < 0.001), visceral pleura invasion (PL1 + 2 versus PL0, DFS, median: 11.40 ± 2.02 versus 25.33 ± 11.55, *p* < 0.001), and advanced stage (stage 3 versus stage 1 + 2, DFS, median: 72.83 ± 29.47 versus 15.60 ± 1.17, *p* < 0.001) manifested a poorer DFS. Conversely, tumor size and Spread Through Air Spaces (STAS) demonstrated no statistically significant impact on DFS.

Patients presenting with lymph node metastasis (with versus without: Overall Survival (OS), median: Non-reach versus 51.80 ± 36.82, *p* < 0.001), higher histologic grade (Grade 1 versus Grade 2 + 3: OS, median, 80.64 ± 4.39 versus 16.00 ± 4.44, *p* < 0.001), visceral pleura invasion (PL1 + 2 versus PL0, OS, median: 52.45 ± 6.49 versus 90.25 ± 4.50, *p* < 0.001), and advanced stage (stage 3 versus stage 1 + 2, OS, median: non-reach versus 51.80 ± 36.82, *p* < 0.001) were associated with a poorer OS. Notably, tumor size (T) and STAS exhibited no statistically significant impact on OS.

**Figure 4:**
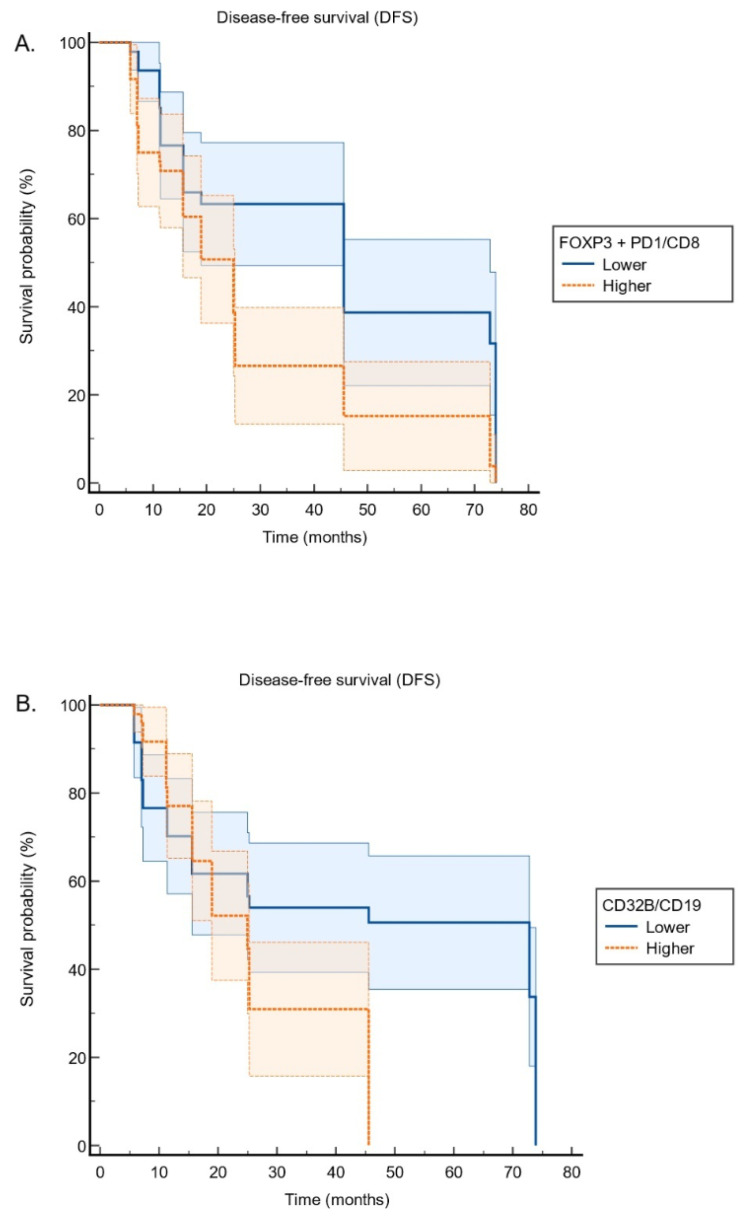
Kaplan–Meier survival curves illustrating the relationship between immune marker expression and survival: (**A**) FOXP3 + PD1/CD8 expression: Higher levels are associated with reduced disease-free survival. (**B**) CD32B/CD19 expression: Patients with higher expression exhibit poorer disease-free survival.

Patients with higher PD-1 expression had significantly shorter DFS compared to those with lower expression (PD-1 expression ≥ 50% vs. < 50%, DFS, median, 5.77 ± 0.0 vs. 15.63 ± 3.64, *p* < 0.001). Similarly, a high FOXP3/CD8 ratio was associated with significantly worse DFS compared to a lower ratio (FOXP3/CD8, higher versus lower: DFS, median: 18.91 ± 1.71 vs. 45.57 ± 8.59, *p* < 0.001). When combining FOXP3 and PD-1 expression relative to CD8 (FOXP3 + PD-1/CD8 ratio), patients with higher ratios exhibited significantly shorter DFS compared to those with lower ratios (FOXP3 + PD-1/CD8 ratio, higher versus lower: DFS, median: 25.00 ± 2.18 vs. 45.57 ± 0.00, *p*= 0.002). (Figure 4A). Furthermore, elevated CD32B/CD19 expression was associated with significantly poorer DFS (CD32B/CD19 ratio, higher versus lower: DFS, median: 25.00 ± 2.62 vs. 72.83 ± 19.92, *p* = 0.009) (Figure 4B). Higher FOXP3/CD8 and PD-1/CD8 ratios were associated with shorter DFS. However, patients with high CD32B/CD19 expression exhibited significantly worse DFS, supporting its role in immune evasion.

The Cox regression analysis examined the association between immune marker expressions and survival outcomes, focusing on two key variables: FOXP3/PD-1/CD8 and CD32B/CD19. The model found that both variables significantly increased the hazard of the event. Specifically, the expression of FOXP3, PD-1, and CD8 markers was associated with a 2.031-fold increase in the hazard (95% CI: 1.255–3.288, *p* = 0.004), suggesting a negative impact on survival. Similarly, increased expression of CD32B and CD19 was linked to a 1.979-fold increase in the hazard (95% CI: 1.162–3.369, *p* = 0.012), indicating a significant adverse effect on survival.

The PD-1 expression has no impact on OS. Higher FOXP3/CD8 ratio (higher versus lower: DFS, median: 69.30 ± 6.86 vs. 84.91 ± 5.41, *p* = 0.044) and higher FOXP3 + PD-1/CD8 ratio (higher versus lower: OS, median: 75.32 ± 6.66 vs. 81.10 ± 5.6, *p* = 0.341) have shorter OS. In addition, the expression of CD32B/CD19 had no impact on OS (higher CD32B/CD19 ratio versus lower CD32B/19: OS, median: 69.53 ± 4.88 vs. 76.79 ± 6.63, *p* = 0.937).

The multivariable logistic regression analysis identified PD-L1 high expression, disease stage, and platinum-based chemotherapy as the key determinants of the DFS. PD-L1 positivity was a strong independent predictor (odds ratio [OR] = 34.94, 95% CI 9.26–131.83, *p* < 0.001), and advanced stage similarly showed a marked increase in risk (OR = 36.51, 95% CI 13.30–100.21, *p* < 0.001). In contrast, platinum-based chemotherapy demonstrated a significant protective effect (OR = 0.051, 95% CI 0.014–0.182, *p* < 0.001). Other variables, including FOXP3/PD-1/CD8, CD32B/CD19, EGFR mutation, and smoking status, were not significantly associated with DFS (all *p* > 0.05). In the multivariable logistic regression analysis for OS, higher CD32B/CD19 expression was associated with poorer prognosis (OR = 4.00, 95% CI 1.61–10.00, *p* = 0.002). In contrast, the presence of an EGFR mutation markedly reduced the risk of mortality (OR = 0.11, 95% CI 0.03–0.40, *p* = 0.001), and receipt of platinum-based chemotherapy similarly conferred a significant survival benefit (OR = 0.12, 95% CI 0.03–0.46, *p* = 0.002).

## 4. Discussion

The findings from our study underscore the importance of a comprehensive analysis of the tumor microenvironment and immune marker expressions, are consistent with an immunosuppressive tumor microenvironment and raise hypotheses about how FOXP3, PD 1 and CD32B relate to disease progression, but do not establish causal mechanisms. Our findings suggest that FOXP3, PD 1, and CD32B are candidate prognostic markers that warrant evaluation in larger, prospective cohorts before any clinical application is considered. Future research should aim to further elucidate the roles of these markers and integrate them into personalized treatment approaches to improve patient outcomes in lung adenocarcinoma.

Tumor size and pathological staging were crucial prognostic factors. The predominant histological subtype identified in our study was acinar, followed by papillary, solid, bronchioloalveolar, and mucinous subtypes. Histological grading indicated that the majority of tumors were graded as G2 and followed by G3 constituting. Grade 3 tumor was associated with aggressive features (tumor size, lymph node metastasis, stage, lymphovascular invasion, and pleural invasion). Recurrence-free survival and overall survival were well-stratified according to tumor grade. Most grade 3 tumors exhibit a solid nodular pattern [32]. Therefore, grade 3 tumors could be identified as significant preoperative predictive factors of lung cancer. Histologic types are correlated with prognosis in lung adenocarcinoma. The acinar/papillary type is most common. Survival analysis showed that spread through air spaces (STAS) and PD-L1 expression were independent poor prognostic factors of recurrence-free survival and overall survival in the acinar/papillary cohort [33].

The presence of Spread Through Air Spaces (STAS) was identified in 57.2% of cases, which is indicative of its importance in the progression and metastasis of lung adenocarcinoma. Disease progression data showed that 47.6% of patients experienced recurrence or metastasis, while 28.5% succumbed to the disease. STAS has been introduced as a novel mechanism of invasion that is important for pathologists to recognize [34,35]. There is a compelling body of evidence associating the presence of STAS with lower survival and suggesting that STAS is an independent prognostic factor, regardless of the stage of tumor. These survival metrics underscore the aggressive nature of lung adenocarcinoma and the critical need for effective therapeutic strategies.

The detailed analysis of immune markers within the tumor microenvironment, particularly the expressions of FOXP3, PD-1, and CD32B, offers valuable insights into their potential as prognostic biomarkers and therapeutic targets [36,37]. The correlation between higher expressions of FOXP3 and PD-1 with advanced pathological stages and poorer survival outcomes highlights their role in the immunosuppressive tumor microenvironment. The findings from this study highlight the significant role that immune markers, such as FOXP3, PD-1, and CD32B, play in the tumor microenvironment (TME) of lung adenocarcinoma, providing valuable insights into their potential as prognostic biomarkers and therapeutic targets.

The expression of cytotoxic T-cells (CD8) relative to CD3 was significantly higher in lower histological grades, suggesting a more robust immune response in early-stage tumors. Interestingly, the expression of CD8/CD3 was also significantly associated with the absence of tumor spread through air spaces (STAS), reinforcing the notion that tumors lacking STAS may retain better immune surveillance. However, as cancer progressed with increasing tumor size, lymph node involvement, and visceral pleural invasion, a decreasing trend in CD8/CD3 expression was noted, although these differences were not statistically significant. This may indicate that as the tumor evades immune surveillance, cytotoxic T-cell activity diminishes, potentially contributing to tumor progression [38].

FOXP3, a marker of regulatory T cells (Tregs), showed a significant increase in expression with advancing cancer stages and in cases with lymph node metastasis and visceral pleural invasion. This suggests a role for Tregs in immune evasion, with higher FOXP3 expression contributing to a more immunosuppressive microenvironment that allows tumor progression. The significant correlation between higher PD-1 expression and advanced cancer stages, larger tumor sizes, and lymph node metastasis supports this view, indicating potential immune exhaustion as tumors progress. However, single-marker IHC staining limits our ability to definitively attribute PD-1 expression to specific T-cell subsets, such as CD8+ or CD4+ cells. These findings are in line with previous studies that have demonstrated the role of PD1 in impairing cytotoxic T-cell function in the context of an immunosuppressive TME [39].

The analysis of CD32B, a marker of inhibitory B-cell signaling, revealed a significant increase in its expression relative to CD19 as tumors progressed [40,41]. Higher CD32B/CD19 ratios were observed in advanced stages and in T3 and T4 tumors, pointing toward a possible mechanism of immune evasion through B-cell-mediated inhibitory pathways. This suggests that CD32B may contribute to tumor progression by dampening the immune response, and its elevated expression correlates with worse disease-free survival (DFS). The significant difference in CD32B/CD19 expression between histological grades also supports the hypothesis that higher CD32B expression in lower-grade tumors may suppress immune activation in the early stages of tumor development, while more aggressive tumors show a reduced difference in expression.

Patients with higher expressions of FOXP3, PD-1, and CD32B/CD19 were found to have significantly worse DFS. The Cox regression analysis further validated these findings, showing that increased expression of FOXP3, PD-1, and CD32B/CD19 significantly raised the hazard of poor outcomes. These markers appear to play a critical role in promoting an immunosuppressive environment that allows tumors to grow unchecked and escape immune destruction [42,43]. Conversely, the lack of correlation between PD-1 expression and overall survival (OS) suggests that other factors within the immune landscape may have a more prominent role in influencing long-term outcomes in lung adenocarcinoma.

In this pilot cohort, higher composite FOXP3 + PD-1/CD8 and CD32B/CD19 ratios were each associated with approximately a two-fold increase in the hazard of disease recurrence on Cox analysis, and patients in the higher-ratio groups generally showed shorter DFS, suggesting that a T-cell/Treg-skewed and inhibitory B-cell milieu may favor early relapse in resectable lung adenocarcinoma [36,37,40,41]. At the same time, PD-1 expression alone did not influence OS, FOXP3- and FOXP3 + PD-1-based ratios showed only modest trends toward worse OS, and CD32B/CD19 did not affect OS in unadjusted analyses, indicating that these immune readouts may be more closely linked to early events than to long-term survival. When key clinical factors were incorporated into multivariable logistic regression, PD-L1 positivity and advanced stage emerged as the strongest adverse determinants of DFS, whereas platinum-based chemotherapy conferred a marked protective effect, consistent with the established prognostic impact of PD-L1 expression, stage, and adjuvant treatment in NSCLC [32,33,34,35]. In this context, neither FOXP3 + PD-1/CD8 nor CD32B/CD19 remained independently associated with DFS, whereas higher CD32B/CD19 did retain an association with poorer OS, while EGFR mutations and platinum-based therapy were each linked to improved survival, underscoring the dominant influence of tumor stage, molecular profile, and systemic treatment on outcome. Overall, these results support the concept that FOXP3-, PD-1-, and CD32B-related immune phenotypes capture an immunosuppressive tumor microenvironment, but in this small, retrospective study their prognostic contribution appears secondary to major clinicopathologic and therapeutic variables and should be interpreted as hypothesis-generating.

This single-center, retrospective pilot in resectable lung adenocarcinoma enrolled 21 patients with heterogeneous stages, among whom 10 experienced disease progression and 6 died during follow-up, limiting power, particularly for OS, and increasing the risk of overfitting and residual confounding despite parsimonious adjustment. Given the small, heterogeneous cohort and retrospective design, all reported associations between immune markers and outcomes should be interpreted as exploratory; they cannot distinguish cause from consequence or support immediate changes in clinical management. Estimates should therefore be viewed as hypothesis-generating: multiple clinicopathologic comparisons in a small dataset raise the possibility of type I error, and any dichotomized cut-points or Kaplan–Meier displays are provided for visualization rather than inference. The study’s field-based sampling (five 200× ROIs per stain) and area-threshold quantification may incompletely capture intratumoral heterogeneity; area-based metrics can diverge from cell densities, and ratio measures (e.g., FOXP3/CD8, CD32B/CD19) can be unstable when denominators are low. Single-marker IHC also precludes definitive cellular attribution (e.g., PD-1 to CD8+ vs. other T-cell subsets; CD32B to specific B-cell lineages) and limits spatial context (e.g., proximity to effector cells or tertiary lymphoid structures). Potential pre-analytic and analytic variation (fixation, antigen retrieval, clone-specific performance, inter-run effects) and the absence of formal inter/intra-observer reproducibility assessments introduce measurement error. As a single-institution cohort, selection and treatment confounding are possible (e.g., unmodeled adjuvant therapy, smoking and comorbidity profiles), which may affect generalizability beyond resectable, surgery-treated cases. Future work should validate these findings in larger, stage-balanced cohorts; standardize pre-analytics and run controls; include blinded reproducibility studies; adopt cell-level digital quantification (with macro/code sharing); employ multiplex/spatial profiling to confirm cellular identity and neighborhood relationships; and integrate oncogenic drivers (e.g., EGFR, KRAS, ALK) to clarify interactions between genotype and immune phenotype before any clinical implementation.

## 5. Conclusions

In conclusion, this study highlights the prognostic significance of FOXP3, PD-1, and CD32B expression in resectable lung adenocarcinoma, emphasizing their combined impact on patient survival outcomes. Elevated FOXP3 and PD-1 expressions correlate significantly with advanced disease stages and poorer prognoses, highlighting their central roles in immune suppression and tumor progression. Similarly, CD32B expression has been identified as a critical mediator of immune evasion. These data motivate further mechanistic and translational studies to test whether modulating these pathways could have therapeutic relevance, our study alone is insufficient to guide treatment decisions. Comprehensive molecular and immunohistochemical evaluations of the tumor microenvironment will be essential for refining prognostic assessments and developing personalized therapeutic strategies in lung adenocarcinoma.

In summary, this small, retrospective pilot suggests that higher FOXP3, PD 1, and CD32B (relative to CD8 or CD19) levels are associated with adverse pathological features and shorter DFS in resectable lung adenocarcinoma. These findings are hypothesis generating and require confirmation in larger, prospective, stage-balanced cohorts before they can inform clinical decision making.

## Figures and Tables

**Table 1:** Clinical and Pathological Findings of Lung Cancer Patients.

Characteristic	*N* (%)
**Age (years old)**	
median (range)	66 (41–86)
**Gender**	
Female	12 (57.1)
Male	9 (42.9)
**Smoking status**	
Current or former	10 (47.6)
Never	11 (52.4)
**Tumor size**	
≤3 cm	7 (33.3)
>3 cm	14 (66.7)
**Pathological stage**	
Stage I-II	7 (33.3)
Stage IIIA	8 (38.1)
Stage IIIB	6 (28.5)
**Predominant subtype**	
Acinar	16 (76.1)
Papillary	1 (4.8)
Solid	2 (9.5)
Bronchioloalveolar	1 (4.8)
Mucinous	1 (4.8)
**Pleural invasion (PL)**	
PL0	13 (61.9)
PL1	5 (23.8)
PL2	3 (14.3)
**Histological Grade**	
G1	1 (4.8)
G2	14 (66.7)
G3	6 (28.5)
**Spread Through Air Spaces (STAS)**	
Not identified	5 (23.8)
Indeterminate	4 (19.0)
Present	12 (57.2)
**Mutation type**	
EGFR (exon 19 deletion + L858R)	11 (52.4)
ERBB2	2 (9.5)
Wild type	4 (19.0)
Not detected	4 (19.0)
**PD-L1 expression**	
<1%	9 (42.9)
1–49%	5 (23.8)
≥50%	1 (4.8)
Not detected	6 (28.6)
**Adjuvant treatment**	
Platinum-based chemotherapy	11 (52.4)
Tegafur/Uracif	3 (14.3)
Tyrosine-kinase inhibitor	3 (14.3)
None	4 (19.0)
**Disease progression**	
Recurrence or metastasis	10 (47.6)
Death	6 (28.5)
**Survival (months), median (range)**	
DFS (Disease free survival)	18.97 (5.77–73.9)
OS (Overall survival)	25.20 (7.03–106.63)

**Table 2:** Tumor Microenvironment Evaluation through Immunohistochemical Staining.

	CD3	CD8	FOXP3	PD1	CD19	CD32B
T						
T1 (*n* = 7)	29.18 ± 11.47	29.81 ± 10.71	8.97 ± 6.12	20.74 ± 14.89	25.13 ± 16.12	28.39 ± 9.10
T2 (*n* = 8)	26.07 ± 8.72	29.48 ± 7.22	11.87 ± 5.57	22.14 ± 7.43	22.22 ± 8.85	31.76 ± 7.53
T3 +T4 (*n* = 6)	31.02 ± 8.29	27.03 ± 11.14	11.81 ± 7.58	27.49 ± 8.74*	22.02± 5.63	32.20 ± 11.12
N						
N0 (*n* = 9)	24.20 ± 4.61	26.76 ± 4.06	7.38 ± 4.30	20.26 ± 5.49	27.28 ± 5.13	23.82 ± 3.97
N1 (*n* = 6)	30.34 ± 12.54	27.45 ± 12.13	12.71 ± 6.26	22.41 ± 10.48	23.85 ± 14.30	30.53 ± 9.25
N2 (*n* = 6)	27.79 ± 7.77	30.35 ± 7.96	14.77 ± 9.42*	24.43 ± 13.06	22.234 ± 9.76	30.68 ± 9.60
Stage						
I -II (*n* = 7)	31.27 ± 11.23	28.90 ± 11.90	9.21 ± 5.71	17.29 ± 9.82	28.32 ± 10.87	27.71 ± 8.95
IIIA (*n* = 8)	25.99 ± 8.24	27.87 ± 7.83	10.20 ± 5.94	26.99 ± 10.49	22.01 ± 2.52	29.97 ± 9.29
IIIB (*n* = 6)	28.88 ± 8.05	34.06 ± 5.23	19.72 ± 4.15*	33.08 ± 5.84*	18.99 ± 11.61	32.22 ± 10.07
Histology grade (G)						
G1 (*n* = 1)	20.04 ± 10.34	22.86 ± 1.94	2.44 ± 1.32	4.71 ± 2.16	25.58 ± 6.01	24.17 ± 2.91
G2 (*n* = 14)	29.06 ± 10.69	29.86 ± 10.60	10.35 ± 5.26	19.78 ± 6.68	23.80 ± 12.40	30.39 ± 10.71
G3 (*n* = 6)	28.86 ± 7.29	28.16 ± 8.05	11.69 ± 6.73*	26.19 ± 11.68*	2.46 ± 1.03*	32.36 ± 7.35
STAS						
Yes (*n* = 12)	30.73 ± 11.04	30.57 ± 10.27	11.01 ± 5.52	27.36 ± 10.74*	19.89 ± 11.45*	32.85 ± 11.34*
No (*n* = 9)	26.06 ± 7.68	27.16 ± 8.71	10.53 ± 7.37	18.26 ± 9.93	26.25 ± 10.82	28.27 ± 6.52
Visceral pleural invasion (PL)						
PL0 *(n* = 13)	30.84 ± 10.40	30.05 ± 10.19	8.94 ± 5.13	18.25 ± 5.42	24.22 ± 13.57	25.78 ± 10.14
PL1 (*n* = 5)	23.58 ± 6.71	28.91 ± 7.59	10.27 ± 6.31	23.26 ± 13.17	22.28 ± 5.29	31.65 ± 7.31
PL2 (*n* = 3)	28.24 ± 9.49	25.02 ± 10.30	13.02 ± 7.01	25.42 ± 8.39	21.64 ± 9.10	32.64 ± 9.26*

*p < 0.05.

## Data Availability

All data generated or analyzed during this study are included in this published article and Supplementary Materials. Further inquiries can be directed to the corresponding author.

## Supplementary Materials

The following supporting information can be downloaded at: https://www.mdpi.com/article/10.3390/cancers17233886/s1, Figure S1: Representative hematoxylin and eosin (HE) and immunohistochemical staining for T-cell and B-cell markers in resected lung adenocarcinoma.

## Abbreviations

The following abbreviations are used in this manuscript:

CD32B (FCGR2B)	Fc Gamma Receptor IIb
CTL	Cytotoxic T lymphocyte
CTLA-4	Cytotoxic T lymphocyte-associated protein 4
DFS	Disease-Free Survival
FOXP3	Forkhead Box P3
IHC	Immunohistochemistry
PD-1	Programmed Death-1
OS	Overall Survival
STAS	Spread Through Air Spaces
TIL	Tumor-infiltrating lymphocytes
TME	Tumor Microenvironment
Tregs	Regulatory T Cells
