# Supplementary material for: Exploratory Immunohistochemical Profiling of FOXP3, PD-1 and CD32B in Resectable Lung Adenocarcinoma
Source: Cancers (Basel). 2025 Dec 4;17(23):3886. doi: 10.3390/cancers17233886 (PMC12691456; doi:10.3390/cancers17233886)
Supplement: Supplementary file 1 [file cancers-17-03886-s001.zip › cancers-3979848-supplementary.pdf]

Supplementary Materials: Exploratory Immunohistochemical Profiling of FOXP3, PD-1 and CD32B in Resectable Lung Adenocarcinoma

Long-Wei Lin, Hong-Jing Chuang, Kuan-Hsun Lian, Yu-Ting Tseng and Chung-Yu Chen

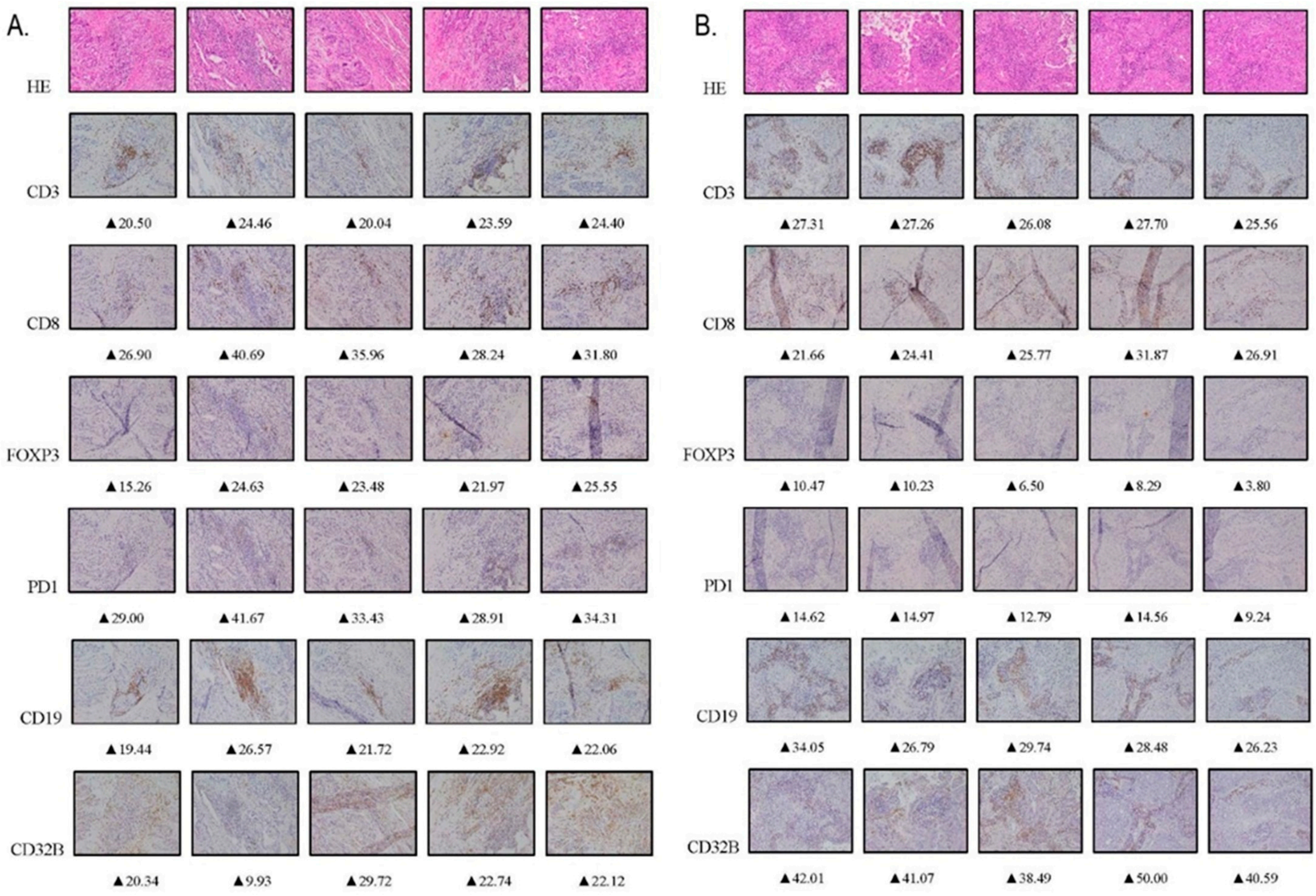

Figure S1. Representative hematoxylin and eosin (HE) and immunohistochemical staining for T-cell and B-cell markers in resected lung adenocarcinoma. For two illustrative cases (A and B), five systematically sampled tumor fields at 200× magnification are shown for HE, CD3, CD8, FOXP3, PD-1, CD19 and CD32B. The black triangles (▲) under each panel indicate the quantitative percentage-positive area (%) for that field as derived from the ImageJ/IHC Profiler pipeline.
